# How does the cladoceran *Daphnia pulex* affect the fate of *Escherichia coli* in water?

**DOI:** 10.1371/journal.pone.0171705

**Published:** 2017-02-08

**Authors:** Jean-Baptiste Burnet, Tarek Faraj, Henry-Michel Cauchie, Célia Joaquim-Justo, Pierre Servais, Michèle Prévost, Sarah M. Dorner

**Affiliations:** 1 Canada Research Chair in Source Water Protection, Department of Civil, Geological and Mining Engineering, Polytechnique Montreal, Montreal, Quebec, Canada; 2 Environmental Research and Innovation, Luxembourg Institute of Science and Technology, Esch-sur-Alzette, Luxembourg; 3 Laboratoire d’Écologie Animale et d’Écotoxicologie, Institut de Chimie, Université de Liège, Liège, Belgium; 4 Écologie des Systèmes Aquatiques, Université Libre de Bruxelles, Campus de la Plaine, CP 221, Boulevard du Triomphe, Bruxelles, Belgium; 5 NSERC Industrial Chair on Drinking Water, Department of Civil, Geological and Mining Engineering, Polytechnique Montreal, Montreal, Quebec, Canada; University of Illinois at Urbana-Champaign, UNITED STATES

## Abstract

The faecal indicator *Escherichia coli* plays a central role in water quality assessment and monitoring. It is therefore essential to understand its fate under various environmental constraints such as predation by bacterivorous zooplankton. Whereas most studies have examined how protozooplankton communities (heterotrophic nanoflagellates and ciliates) affect the fate of *E*. *coli* in water, the capacity of metazooplankton to control the faecal indicator remains poorly understood. In this study, we investigated how the common filter-feeding cladoceran, *Daphnia pulex*, affects the fate of *E*. *coli* under different experimental conditions. *Daphnia* ingested *E*. *coli* and increased its loss rates in water, but the latter rates decreased from 1.65 d^-1^ to 0.62 d^-1^ after a 1,000-fold reduction in *E*. *coli* initial concentrations, due to lower probability of encounter between *Daphnia* and *E*. *coli*. The combined use of culture and PMA qPCR (viability-qPCR) demonstrated that exposure to *Daphnia* did not result into the formation of viable but non-culturable *E*. *coli* cells. In lake water, a significant part of *E*. *coli* population loss was associated with matrix-related factors, most likely due to predation by other bacterivorous biota and/or bacterial competition. However, when exposing *E*. *coli* to a *D*. *pulex* gradient (from 0 to 65 ind.L^-1^), we observed an increasing impact of *Daphnia* on *E*. *coli* loss rates, which reached 0.47 d^-1^ in presence of 65 ind.L^-1^. Our results suggest that the filter-feeder can exert a non-negligible predation pressure on *E*. *coli*, especially during seasonal *Daphnia* population peaks. Similar trials using other *Daphnia* species as well as stressed *E*. *coli* cells will increase our knowledge on the capacity of this widespread zooplankter to control *E*. *coli* in freshwater resources. Based on our results, we strongly advocate the use of natural matrices to study these biotic interactions in order to avoid overestimation of *Daphnia* impact.

## Introduction

Faecal contamination of freshwater is traditionally assessed through the enumeration of faecal indicator bacteria (FIB) such as *Escherichia coli*. Monitoring of FIB in recreational and/or drinking water resources drives the implementation of mitigation measures to protect public health from microbial risks [1,2]. Considering the central role of *E*. *coli* in microbial water quality assessment, it is essential to understand its fate in aquatic habitats. Temperature, low nutrient levels and solar irradiation [3,4], as well as microbial competition [5] can affect *E*. *coli* survival in water. Also, predation by indigenous biota is another major driver of its fate [6–9]. Several studies have addressed the impact of protozooplankton communities of heterotrophic nanoflagellates (HNF) and ciliates on *E*. *coli* [10,11] because they are major consumers of bacterioplankton in aquatic ecosystems [12,13]. By comparison, less is known on the extent to which metazooplankton communities may affect the fate of *E*. *coli* in natural waters [14]. Cladocerans (or “water fleas”) regroup cosmopolitan communities of freshwater microcrustaceans that are important components of aquatic food webs [15]. Among cladocerans, the filter-feeding species of the genus *Daphnia* are able to ingest pelagic food particles (including bacteria) over a wide range of sizes by collecting them with their thoracic appendages [16–18] and they can collectively filter considerable volumes in only short periods of time. As a keystone species, *Daphnia* can affect the biomass of aquatic microbial communities and shape their size structure and species composition, either by direct consumption of bacteria or indirectly by predation on bacterivorous nanoflagellates and ciliates [19–23].

Despite the role of cladocerans in the regulation of bacterial populations, limited information is available on their interactions with allochthonous microorganisms introduced in freshwater bodies through faecal pollution. *Daphnia* can ingest and affect the viability of the protozoan pathogens *Cryptosporidium* and *Giardia* [24,25]. Also, *Daphnia carinata* was shown to negatively impact the fate of the bacterial pathogen *Campylobacter jejuni* [26]. Conversely, other studies suggest that *Daphnia* can act as a refuge for ingested faecal microorganisms such as *E*. *coli* and offer them some protection during drinking water treatment [27,28]. To the best of our knowledge though, it is not known to what extent *Daphnia* can affect *E*. *coli* in natural waters. Early studies have determined ingestion and assimilation rates of *E*. *coli* by *Daphnia* using radioactive tracers in synthetic water [29,30], but the technique incurs several methodological limitations and may overestimate removal rates for food particles resistant to digestion [31]. Also, because regulatory monitoring of *E*. *coli* in water is usually performed using culture-based methods, it appears more pertinent to assess *Daphnia* impact through enumeration of the FIB by culture. Importantly though, culture-based methods do not allow the detection of potentially viable but non-culturable (VBNC) cells that may have lost their ability to grow on culture media due to various external stresses [32]. Like many other bacteria, *E*. *coli* can switch to a VBNC state under stressful conditions, which can ultimately result in false-negatives with potential sanitary implications [33]. An alternative molecular-based method called propidium monoazide (PMA) PCR (or viability-PCR) is increasingly used to overcome this limitation [34]. When the bacterial membrane is damaged, PMA enters the cell and binds irreversibly to the DNA, thereby inhibiting PCR amplification and allowing a differentiation between viable and non-viable cells. PMA qPCR relies on the same principle as the *Bac*Light LiveDead assay, PMA being a deritative of propidium iodide (PI), which enters cells with a damaged membrane [35]. By comparing PCR signals from PMA-treated and untreated cells, it is thus possible to calculate the proportion of viable cells in a sample (Fittipaldi et al. 2012 and citations herein).

Considering (i) that *E*. *coli* is used as indicator of faecal pollution in most water regulations and given (ii) the high probability of its co-occurrence with *Daphnia* in freshwater ecosystems as well as (iii) the limited knowledge on its specific interactions with *E*. *coli*, the goal of our study was to investigate if and to what extent *Daphnia* could remove *E*. *coli* from water. For this purpose, we used *Daphnia pulex* as model species to assess the loss rate of *E*. *coli* exposed to various *Daphnia* to *E*. *coli* ratios. Available studies that addressed *Daphnia* grazing on faecal microorganisms used synthetic water matrices [24,26]. In order to extend our observations to natural conditions, we also investigated *E*. *coli* loss rates in lake water using a gradient of *Daphnia* population densities. Finally, we compared PMA-qPCR and culture-based enumeration of *E*. *coli* following exposure to *Daphnia* in order to find out whether or not gut passage could result into the induction of VBNC cells.

## Materials and methods

### 1. Model organisms

*Daphnia pulex* Leydig, 1860 were purchased from Carolina Biology Supply (Burlington, CA). A clonal culture of *D*. *pulex* was maintained in the laboratory at 20°C, grown in artificial *Daphnia* medium (ADaM) [36] and fed with *Nannochloris atomus* obtained from the National Center for Marine Algae and Microbiota (NCMA) during at least 6 months prior to the trials. ADaM medium was prepared by adding 0.333 g.L^-1^ synthetic seasalt (InstantOcean) and the following analytical grade chemicals (Fisher Scientific) to deionized water as described by Klüttgen et al. [36]: CaCl_2_ (117.6 g.L^-1^), NaHCO_3_ (25.2 g.L^-1^) and SeO_2_ (1.4 g.L^-1^). Cultures of *N*. *atomus* were maintained at 20°C in modified Bold’s basal medium (BBM) over 18:6 light-dark cycles and with continuous stirring and air bubbling. After approximately 1 week, algae were harvested by centrifugation (3350 *g*, 10 minutes) and stored at 4°C for daily feeding of *Daphnia*.

Initial microscope observations of *Daphnia* ingestion kinetics were performed using *Escherichia coli* K12 MG1655 strain (ATCC 700926). Microcosm experiments were performed with an environmental *E*. *coli* strain isolated from Missisquoi Bay, a shallow transboundary bay of Lake Champlain straddling the U.S.A/Canada border and described in detail by [37]. Both strains were preserved in TSB-glycerol at -80°C. Before each experiment, a new sub-culture was inoculated on Tryptic Soy Agar (Thermo Fisher Scientific) and incubated at 35°C during 18–20 hours. Cells were harvested, re-suspended in sterile phosphate buffer and adjusted to an OD_600_ of 1.0 (corresponding to ~10^9^ CFU.mL^-1^ as verified by culture). The stock suspension was then quantified by plate counting on TSA using 10^−6^ and 10^−7^ dilutions.

### 2. Observation of *D*. *pulex* feeding *on E*. *coli*

In order to visualize the ingestion of *E*. *coli* by *D*. *pulex*, 5 individuals were incubated in mineral water (Volvic) and fed with 10^6^−10^7^ CFU.mL^-1^ *E*. *coli* (strain MG1655), preliminarily labelled with 4’,6-diaminophenyl-1H-indole-6-carboxamidine (DAPI) at a concentration of 1 μM. During first trials, food boluses containing *E*. *coli* had already reached the distal part of *Daphnia* guts after 30 minutes. As a result, further feeding experiments were performed during shorter incubation periods of 15, 5 and 2 minutes. Following incubation, *D*. *pulex* was narcotized with carbonated water during 1 minute, killed with formaldehyde and mounted on a slide for observation of gut content under an epifluorescence microscope (Olympus, 10x magnification) equipped with a blue excitation filter cube (Olympus, U-MWU, 330–385 nm excitation band).

### 3. Determination of *E*. *coli* loss rates in the presence of a *D*. *pulex* population

#### 3.1. Synthetic matrix

First experiments were carried out to determine *E*. *coli* loss rates in presence and absence of *Daphnia pulex* using bottle-microcosms (1.3 L) filled with ADaM medium (see section 1). *Daphnia* microcosms contained 40 *D*. *pulex* juveniles of similar size (~1 mm) and control microcosms without *D*. *pulex* were run to assess natural *E*. *coli* population losses. *Daphnia* and control microcosms were run in triplicate and incubated on a zooplankton wheel (rotation at 1 rpm during 2 minutes every 2 hours) during 48 hours at 20°C under 18:6 light-dark cycles.

To assess the effect of *E*. *coli* initial concentration on its loss rate, two different spike doses were used to achieve initial concentrations of either 10^3^ or 10^6^ CFU.mL^-1^. A small amount of green algae *Nannochloris atomus* (~7,000 cells.mL^-1^, corresponding to 0.1 mg C.L^-1^) was added to stimulate grazing [26]. To test how the amount of algal food would affect *D*. *pulex* predation on *E*. *coli*, an additional experiment was conducted with microcosms containing *E*. *coli* at 10^3^ CFU.mL^-1^ incubated in presence of high concentrations of *N*. *atomus* (1.3 10^5^ cells.mL^-1^, corresponding to 1.7 mg C.L^-1^).

Immediately after the onset of an experiment, a first sample (100 μL-samples or appropriate dilutions) was collected after manual mixing of the bottle by gentle up and down movements, giving special care to avoid any harm to *Daphnia*. Upon sampling, the bottles were capped with parafilm and incubated on the zooplankton wheel. Sampling was repeated after 24 and 48 hours (T_*24*_, T_*48*_) following the same procedure. Culturable *E*. *coli* were enumerated in each sample following USEPA method 1604 [38]. Samples were added to 50 mL sterile phosphate buffer and filtered on sterile cellulose ester membranes (47 mm diameter, 0.45-μm pore-size) which were then placed on MI agar (BD Biosciences) and incubated at 35°C during 18–24 hours. The loss rate (k) was calculated using the equation Ln(*C*_*t*_/*C*_*0*_) = -kt, where *C*_*0*_ and *C*_*t*_ are the concentrations in culturable *E*. *coli* (CFU.mL^-1^) at T_0_ and T_48,_ respectively, and *t* is the incubation time (days). Loss of *E*. *coli* followed a first order kinetic between 0 and 48 hours as verified by regression analyses (r^2^ ranged between 0.77 and 0.96, p<0.01).

To estimate the rate at which *Daphnia* removed particles from the water, algae were counted every 24 hours using a Neubauer counting chamber for both *Daphnia* and control microcosms that were initially spiked with an algal food concentration of 1.3 10^5^ cells.mL^-1^. After each count, fresh algae from the stock suspension were added (equivalent to the quantity consumed by *Daphnia* during 24 hours) in order to maintain a constant food source throughout the experiment. Calculated removal rates were then used to estimate the theoretical loss rate of *E*. *coli* (i.e. first order kinetic) with the assumption that both *N*. *atomus* (~5 μm) and *E*. *coli* (1–2 μm) were ingested with comparable efficiency and homogenously distributed within the microcosm. In control microcosms, algae were counted to account for any change in cell concentration due to factors others than *Daphnia* feeding.

#### 3.2. Lake water matrix

In a second set of experiments, loss rates of culturable *E*. *coli* were assessed in lake water (Missisquoi Bay, QC, Canada) collected on Sept 1, 2015 (permitted by Aquatech Inc. at Philipsburg drinking water intake) and passed through a 53 μm mesh-size filter to remove metazooplankton species (cladocerans, large rotifers and copepods). Under the same ambient conditions as for synthetic water (temperature of 20°C, intermittent rotation of the bottle-microcosms on a zooplankton wheel), *Daphnia* microcosms were then spiked with 10, 40 or 80 individuals (~1 mm), resulting in final densities of 8, 32 and 65 ind.L^-1^. Two additional sets of control microcosms containing only raw or 53 μm-filtered lake water were run to account for natural loss of culturable *E*. *coli*. *Daphnia* and control microcosms were run in triplicate, spiked with *E*. *coli* to reach a concentration of 10^3^ CFU.mL^-1^ and processed as described above.

### 4. Culturability and viability of *E*. *coli* following *D*. *pulex* grazing

In order to determine if culture effectively accounted for *E*. *coli* mortality following exposure to *Daphnia*, short experiments were performed in ADaM medium containing *E*. *coli* at 10^6^ CFU.mL^-1^ and low amounts of *N*. *atomus* (~7,000 cells.mL^-1^) to stimulate grazing. Incubation was performed during 24 hours in 15 mL-wells (12-well plates, Corning) at 20°C under 18:6 light-dark cycles and in presence of *Daphnia* (~1 mm; 1 individual per well). Additional control wells without *D*. *pulex* were run in parallel. Two independent experiments were carried out, resulting in a total of 4 and 5 control and *Daphnia* wells, respectively. Each well was sampled at the onset of the experiment (T_*0*_) and subsequently after 3, 6, 12 and 24 hours (T_*3*_, T_*6*_, T_*12*_ and T_*24*_, respectively). For each sample, 1 mL was collected from the upper third of the well to avoid re-suspension of faecal deposits, briefly mixed and split for culture-based (100 μL) and qPCR-based (900 μL) quantification.

Culturability of *E*. *coli* was determined by spread-plating 100 μL-samples or appropriate dilutions onto Tryptic Soy Agar followed by incubation at 35°C for up to 24 hours. Cell viability (based on membrane integrity) was evaluated by PMA qPCR by subdividing 900 μL-aliquots into two equal parts either treated with PMA (for quantification of viable cells only) or not treated (for quantification of viable and dead cells). For PMA treatment, aliquots were incubated during 5 minutes with 50 μM PMA (Biotium Inc.) in the dark and at ambient room temperature. Photoactivation was performed during 15 minutes at ambient room temperature using a PMA Lite device (Biotium Inc.). Both treated and untreated cells were harvested by centrifugation (10,000 *g*, 3 minutes) and directly processed for DNA extraction or stored at -20°C until processed. Controls using heat-inactivated *E*. *coli* were performed to verify the performance of PMA in inhibiting the PCR signal from heat-inactivated (dead) cells. DNA from treated and untreated cells was extracted with the DNeasy kit (Qiagen) according to manufacturer instructions and purified DNA was quantified by real-time PCR [39]. Final concentrations of primers and TaqMan probe were found optimal at 300 nmol.L^-1^ and 100 nmol.L^-1^, respectively. PCR was run on a Rotorgene-6000 instrument (Corbett Life Science) with TaqMan Universal Mastermix II (Thermo Fisher Scientific) and 5 μL DNA under the following thermocycling conditions: 10 minutes hold at 95°C followed by 45 cycles including annealing at 95°C during 15 seconds and hybridization/elongation at 60°C during 1 minute. Extraction and PCR blanks were performed as controls and standard curves were added to each real-time PCR assay. Standard curves were constructed using genomic DNA extracted from a pure culture of *E*. *coli* (~10^7^ CFU.mL^-1^) as described above and serially log-diluted in DNase-free water. PCR results are expressed in CFU equivalent per millilitre (CFUeq.mL^-1^).

### 5. Statistical analyses

One-way analyses of variance (ANOVA 1) followed by Tukey’s *post hoc* test were performed to test the significance of differences in *E*. *coli* loss rates under the tested conditions. Least-squares linear regression was performed to analyse the relationship between culturable and viable *E*. *coli* cells as well as to verify that the loss rates followed a first order kinetic. All analyses were run in Statistica v.12 (StatSoft, Inc.). Significance was assessed at a p<0.05 level.

## Results

### 1. Observation of fluorescently-labelled *E*. *coli* cells following *D*. *pulex* ingestion

**Fig 1:**
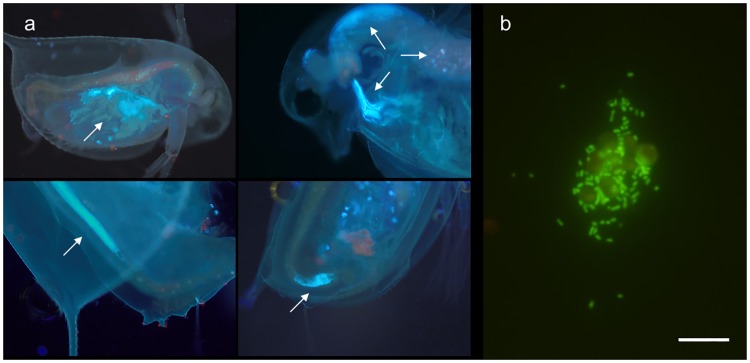
Ingestion and excretion of *Escherichia coli* by *Daphnia pulex*. (a) Gut content analysis of *D*. *pulex* following exposure to DAPI-labelled *E*. *coli* cells. White arrows indicate food boluses with compacted *E*. *coli* cells. Autofluorescence of ingested algae is also visible in some captions and varies from red to brownish, reflecting the stage of digestion of the chlorophyll. (b) Viability of *E*. *coli* following *D*. *pulex* gut passage as seen on an aggregate of *E*. *coli* rods and algal cells of *Nannochloris atomus* (Scale bar = 20 μm).

*Escherichia coli* cell clusters were observed in the guts of all 5 *Daphnia* individuals after 15, 5 and 2 minutes of incubation. Fluorescent *E*. *coli* clusters were localized in the filtering chamber containing the thoracic appendages as well as in the foregut, midgut and hindgut (Fig 1a).

### 2. Removal of *E*. *coli* by *D*. *pulex* in synthetic water

**Fig 2:**
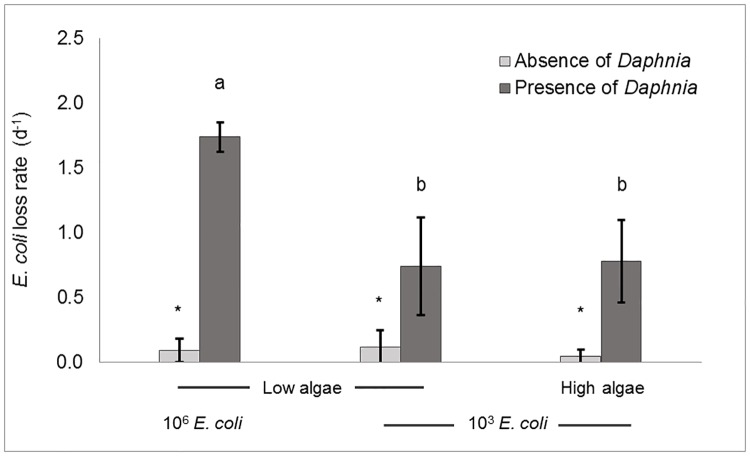
Grazing of *Daphnia pulex* on *Escherichia coli* in ADaM matrix. Effect of *E*. *coli* initial concentration (10^3^ or 10^6^ CFU.mL^-1^) and algae quantity (low algal food, 0.1 mg C.L^-1^ or high algal food, 1.7 mg C.L^-1^) on *E*. *coli* loss rates (in d^-1^) following 48 hours incubation in absence or presence of *D*. *pulex* at densities of 32 ind.L^-1^. The various letters indicate significant (p<0.05) differences in *E*. *coli* loss rates among conditions. Asterisks highlight significant differences in loss rates between presence and absence of *D*. *pulex*.

During the first set of experiments using ADaM medium, concentrations of culturable *E*. *coli* declined significantly (p<0.05) more in presence of *Daphnia pulex* (32 individuals.L^-1^) than in its absence (Fig 2). When spiked at high initial concentration of ~10^6^ CFU.mL^-1^, loss of culturable *E*. *coli* occurred at a rate of 1.74 d^-1^. When spiked at lower concentrations (~3 Log_10_ CFU.mL^-1^), their loss rates decreased to 0.74 d^-1^. In the absence of *D*. *pulex*, *E*. *coli* concentrations always decreased at much lower rates, which did not exceed 0.12 d^-1^ (Fig 2). After subtraction of the loss rates obtained from control microcosms, *Daphnia*-mediated loss in culturable *E*. *coli* reached 1.65 d^-1^ and 0.62 d^-1^ for high and low initial concentrations, respectively. The addition of algal food to the microcosms did not result in a significant change (p>0.05) in average *E*. *coli* loss rates (Fig 2).

### 3. Removal of *E*. *coli* by *D*. *pulex* in lake water

**Fig 3:**
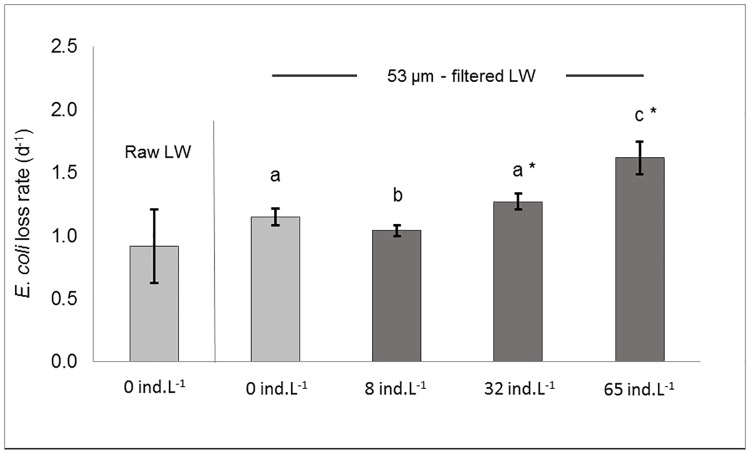
Grazing of *Daphnia pulex* on *Escherichia coli* in a lake water matrix. Loss rates (in d^-1^) of *E*. *coli* are measured following 48 hours incubation in presence of a *D*. *pulex* gradient (8, 32 and 65 ind.L^-1^) in 53 μm-filtered lake water. Filtered and raw lake water samples (light grey) serve as controls to determine *E*. *coli* loss rates in absence of *D*. *pulex*. The letters indicate significant (p<0.05) differences in *E*. *coli* loss rates between *Daphnia* densities in filtered lake water. Asterisks report significant differences between the raw lake water control and filtered lake water samples. LW, lake water.

Lake water metazooplankton was mainly composed of rotifers but also contained cladocerans and copepod nauplii (S1 Table). Flagellates occurred at densities of 868 ind.L^-1^. Loss in culturable *E*. *coli* occurred in absence of *Daphnia* both in raw and filtered lake water (FLW) at similar rates of 0.92 d^-1^ and 1.15 d^-1^, respectively (p > 0.05). In FLW, *E*. *coli* loss rates significantly increased with *D*. *pulex* population densities (Fig 3). In presence of 65 ind.L^-1^ *Daphnia*-mediated loss rates reached 0.47 d^-1^ after subtraction of matrix-related ones.

### 4. Viability and culturability of *E*. *coli* following *D*. *pulex* ingestion

**Fig 4:**
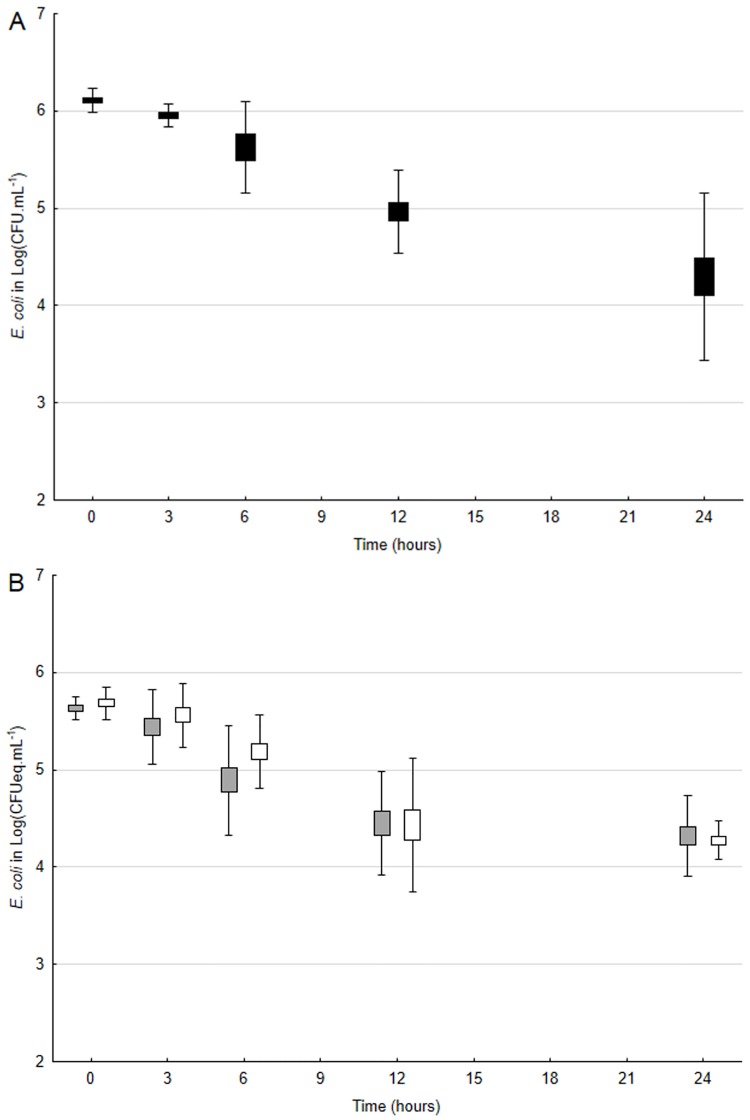
Viability and culturability of *Escherichia coli* upon exposure to *Daphnia pulex*. (a) Box plots (mean ± 2 SD) for (a) culturable (black) as well as (b) total (white) and viable (grey) *E*. *coli* in the presence of *D*. *pulex* (1 individual per 15 mL-well) over 24 hours. CFU, colony forming unit.

**Fig 5:**
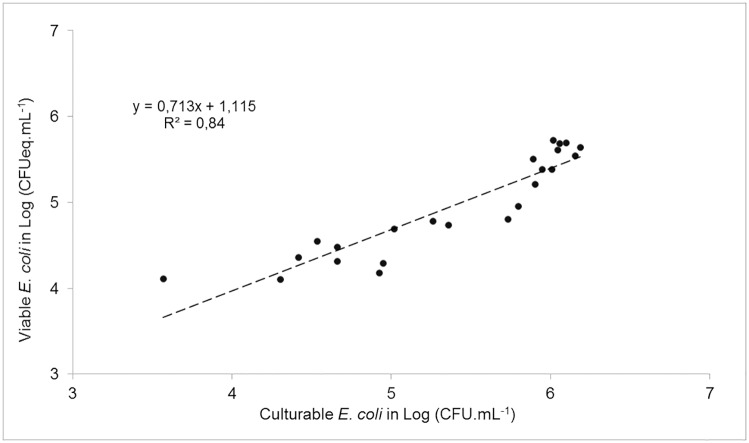
Log-Log plot between culturable and viable *Escherichia coli* cells after 24 hours exposure to *Daphnia pulex*. *E*. *coli* was quantified by culture (CFU.mL^-1^) and PMA qPCR (CFUeq.mL^-1^). CFU, colony forming unit.

In the presence of a single daphnid, average concentrations of culturable *E*. *coli* decreased from 6.1 ± 0.0 Log_10_ CFU.mL^-1^ to 4.3 ± 0.4 Log_10_ CFU.mL^-1^ within 24 hours. As measured by qPCR for untreated and PMA-treated cells, total and viable *E*. *coli* decreased from 5.7 ± 0.1 to 4.3 ± 0.2 Log_10_ CFUeq.mL^-1^and from 5.6 ± 0.1 to 4.3 ± 0.2 Log_10_ CFUeq.mL^-1^, respectively. There were no significant differences (ANOVA 1, p>0.05) between PCR signals generated for PMA-treated and untreated cells over the duration of the experiment (Fig 4). Culturable and viable (PMA-treated cells) were positively correlated (*r*^*2*^ = 0.84, p<0.05) during the 24-hour experiment (Fig 5). In the absence of *D*. *pulex*, average concentrations of culturable, total and viable *E*. *coli* cells did not significantly decrease within 24 hours (ANOVA 1, p>0.05). At T_0_, they were 6.1 ± 0.0, 5.6 ± 0.1 and 5.7 ± 0.2 Log_10_ CFUeq.mL^-1^, respectively, while at T_24_, they were 6.0 ± 0.1, 5.3 ± 0.2 and 5.0 ± 0.5 Log_10_ CFUeq.mL^-1^, respectively. Resulting loss rates of culturable *E*. *coli* were 4.2 ± 0.9 d^-1^ in the presence of one *D*. *pulex* individual.

## Discussion

Bacterivory by higher organisms such as *Daphnia* is a major driver of bacterioplankton abundance and composition in freshwater bodies and it has been extensively studied by limnologists and microbial ecologists on natural bacteria in ponds, lakes and reservoirs [18–20,40]. However, comparatively less is known about the impact of *Daphnia* on faecal microorganisms such as *E*. *coli* despite the widespread occurrence of the cladoceran in lakes and reservoirs contaminated by faecal pollution. Since regulatory monitoring of faecal pollution is performed by culture-based enumeration of *E*. *coli* in water, it is thus essential to understand how *Daphnia* influences the fate of the faecal indicator in water. In the present study, we therefore examined how *Daphnia* affects the culturability and viability of *E*. *coli* in water and we determined its loss rates under various *Daphnia* exposure conditions including lake water containing natural biotic communities.

### Ingestion of *E*. *coli* by *Daphnia pulex*

First exposure experiments involved *E*. *coli* and *Daphnia* in synthetic water and showed that the cladoceran ingested *E*. *coli* within minutes after its addition to the medium. All individuals contained fluorescent *E*. *coli* cell clusters in their guts and after 2 minutes, *E*. *coli* had already reached the second half of the midgut in some individuals (Fig 1a). Ingestion of food by *Daphnia* is not selective and mainly driven by the size of food particles [41]. We used logarithmic-phase *E*. *coli* cells from nutrient-rich growth media that measure about 1–2 μm and fit within the size range of food that can be captured by *Daphnia*. In surface water, *E*. *coli* cells are expected to be smaller due to stresses such as nutrient scarcity, but experimental data from Brendelberger et al. [16] report a retention efficiency of 80% for the smallest bacterioplankton (<1 um). This suggests that *D*. *pulex* would be able to efficiently collect *E*. *coli* in surface water. In addition, bacterial uptake can be increased by “piggybacking”, a phenomenon during which the filtering limbs clog due to the accumulation of food particles [42]. During our feeding experiments, DAPI-labelled *E*. *coli* cells were rapidly ingested by *D*. *pulex* with limited inter-individual variability in gut fullness. Also, smaller individuals (<1 mm) did readily ingest *E*. *coli* (Fig 1a). In natural populations, *Daphnia* size distribution and associated feeding patterns may be more heterogeneous since filtration rates vary with body size and temperature [42]. Interestingly, fluorescent clusters of *E*. *coli* cells were regularly observed in the hindgut of some individuals (Fig 1a), even after 2 minutes incubation only. This observation could indicate the existence of anal water uptake, a mechanism through which *Daphnia* pumps water into the hindgut [43]. Although this behaviour was shown to contribute to nanoparticles uptake in *Daphnia* [44], it is not known if and to what extent it can affect *E*. *coli*.

### *Daphnia* impact on *E*. *coli* culturability and viability

The impact of *Daphnia* on *E*. *coli* loss rates was described in synthetic water in order to compare with previous grazing studies on *Campylobacter* and protozoan parasites [24,26]. After 24 hours, exposure to *D*. *pulex* already resulted in a significant loss in *E*. *coli* population in the microcosm (Fig 2). Using initial *E*. *coli* concentrations of 10^6^ CFU.mL^-1^, we measured *Daphnia*-mediated loss rates of 1.65 d^-1^ after subtraction of natural losses obtained from control microcosms, which corresponds to a net *E*. *coli* loss of 1.4 Log_10_ (i.e. 81% of the initial *E*. *coli* stock) within 48 hours. Under similar experimental conditions (synthetic water matrix, 40 ind.L^-1^), Schallenberg et al. [26] obtained comparable results for *Daphnia carinata* feeding on *Campylobacter jejuni* with a 1.5–2 Log_10_ net removal after 48 hours. However, at lower *E*. *coli* concentrations of 10^3^ CFU.mL^-1^_,_ we observed that the loss rates decreased to 0.62 d^-1^ (removal of 54% of the initial *E*. *coli* stock) (Fig 2). This is less than twice the rates measured with 1,000 higher *E*. *coli* initial concentrations and reflects the lower probability of encounter between *Daphnia* and *E*. *coli*. Using the initial concentration of 10^3^ CFU.mL^-1^, we further evaluated how the addition of algae would affect *E*. *coli* loss rates. These remained unchanged (0.74 and 0.78 d^-1^) in the presence of low or high algal biomass (0.1 and 1.7 mg C.L^-1^, respectively) (Fig 2). Similarly, Tezuka [45] concluded that *Daphnia longispina* feeding rate on bacteria was not affected by the presence of algae. Also, assimilation of bacteria by the cladoceran *Ceriodaphnia reticulata* remained unchanged when algae were included in the bacterial diet [46]. The reason for similar *E*. *coli* loss rates in presence of low and high algal food amounts may be due to a combination of feeding behaviour and resistance of *E*. *coli* to gut passage. Low algae concentrations such as those used in our experiments (7,000 cells.mL^-1^) could have forced *D*. *pulex* to reduce filtration efforts in order to save energy costs and consequently ingest fewer bacteria over time. In contrast, the high algal concentrations maintained throughout the experiment at > 10^5^ cells.mL^-1^ could have provided sufficient food for *Daphnia* to continuously ingest *E*. *coli*. However, given the higher nutritive value of green algae compared to *E*. *coli* [47], assimilation of *E*. *coli* may have been suboptimal and culturability preserved upon gut passage.

This hypothesis is supported by the large discordance between observed and theoretical *E*. *coli* removal that we calculated using algal counts. *Daphnia pulex* removed an average of 6.6 ± 1.2 10^7^ algae.d^-1^ from each microcosm that corresponds to an ingested volume of 0.7 ± 0.1 mL.ind^-1^.hour^-1^, which is in the range of typical filtration rates for *D*. *pulex* [42]. As such, a total of 9.10^5^ CFU would have theoretically been ingested by *D*. *pulex* over 48 hours, which means that the entire initial *E*. *coli* pool should have transited through the gut. Although our calculation is based on several assumptions (filtration rate was constant over time, ingested algal cells were all digested), this large discordance suggests that culturability was maintained upon gut passage for at least part of the ingested *E*. *coli* population. Resistance of indigestible or low-quality food cells to gut passage in *Daphnia* has been demonstrated for lake bacteria [48]. Using fluorescent viability dyes, we showed that the excreted *E*. *coli* cells had intact membranes, indicating that they were still viable (Fig 1b). Over time, resistance to gut passage could have been gradually reduced following successive gut passages, given that numerous faecal aggregates were in the size range of edible particles [41]. It is therefore not excluded that *Daphnia* re-ingested *E*. *coli* cells that had already transited across the gut, especially since coprophagy has been reported for *D*. *pulex* [49]. Also, Connelly et al. [24] hypothesized that apparent mechanical disruption of *Giardia* cell wall could have resulted from successive gut passages. In natural settings, it is conceivable that *E*. *coli* cells having survived gut passage are re-ingested by other *Daphnia* individuals, especially during population blooms that occur seasonally in temperate freshwater bodies [19,20].

Since we used culture to assess *E*. *coli* loss rates, we wanted to find out if, due to stresses encountered in the gut, viable but non-culturable (VBNC) *E*. *coli* cells were excreted by *Daphnia*. In a study on the fate of *Enterococcus* upon exposure to sunlight in water, Walters et al. [50] suggested the existence of VBNC cells following their observation that DNA was still detected while culturable cells were not. To test for potential induction of VBNC cells, a feeding experiment was performed to assess both culturability and viability of *E*. *coli* over time using culture and PMA-qPCR (viability-qPCR), respectively. The large majority of *E*. *coli* cells remaining in the water during the experiment was viable given the very similar (p>0.05) results between untreated and PMA-treated cells (Fig 4). Furthermore, these viable cells followed the same trend as culturable ones (Fig 5), implying the apparent absence of VBNC cells after exposure to *Daphnia*. Taken together, our results for synthetic water thus highlight the significant impact of *Daphnia* on the fate of *E*. *coli*.

### *Daphnia* removal of *E*. *coli* from lake water

In natural water resources, it is expected that the impact of *Daphnia* on *E*. *coli* is influenced by complex interactions among local biota. In particular, bacterivorous protozooplankton is known to significantly contribute to bacterial loss, while at the same time, it can be predated by metazoan grazers such as *Daphnia* [12,40,51]. We therefore repeated our microcosm experiments using freshwater collected from the eutrophic shallow Missisquoi Bay, Canada in order to evaluate to what extent *D*. *pulex* impacted *E*. *coli* in the presence of local biota. As shown in raw and filtered lake water controls (Fig 3), *E*. *coli* displayed a non-negligible natural loss rate (~1.0 d^-1^) in absence of *D*. *pulex* after 48 hours. Metazooplankton populations were dominated by rotifers and small cladocerans (S1 Table) but they had no or very little impact on *E*. *coli* as evidenced by similar loss rates between raw and 53-μm filtered lake water. Therefore, *E*. *coli* natural losses were likely due to a combination of protist grazing, bacterial competition, temperature and nutrient scarcity [3–5,9,10]. The abundance of heterotrophic nanoflagellates (HNF) (S1 Table) was in the range of those found in other eutrophic freshwater lakes, where they can be major predators of bacterioplankton [22,51]. Protozooplankton (especially HNF) has been shown to account for up to 90% of *E*. *coli* mortality in river water, with loss rates ranging between 0.2 and 0.8 d^-1^ [10].

In the presence of *D*. *pulex*, the loss rate of *E*. *coli* increased with *Daphnia* population density and peaked at 1.6 d^-1^ in the presence of 65 ind.L^-1^ (Fig 3). Interestingly, *E*. *coli* loss rates were significantly lower in the presence of 8 ind.L^-1^ than in the absence of *D*. *pulex*. This could be due to the removal of protozooplankton bacterivores by *Daphnia*, which in turn limited their predation pressure on *E*. *coli* [19]. At the same time, *Daphnia* may have been at too low densities to compensate for a decrease in protozooplankton bacterivory. However, at 32 ind.L^-1^, *Daphnia* was numerous enough to exert a predation pressure on both *E*. *coli* and protists. Degans et al. [19] concluded that, at densities of 30 ind.L^-1^, *Daphnia magna* was able to control HNF and ciliates, thereby becoming the dominant bacterivore. An additional explanation for the non-linear increase of *E*. *coli* loss rate with *D*. *pulex* densities may be related to crowding effects, known to occur above 30 ind.L^-1^ [52,53]. This hypothesis is supported by the fact that the individual *D*. *pulex* contribution to *E*. *coli* loss rates progressively decreased from 0.13 to 0.04 and 0.02 d^-1^.ind^-1^ in presence of 8, 32 and 65 ind.L^-1^, respectively.

Although *Daphnia* densities tested in the present study are representative of those found in many freshwater habitats, populations can seasonally peak above 100 and even exceed 1,000 ind.L^-1^ [54–56]. Particularly high population densities (>500 ind.L^-1^) have also been reported from aerated sewage ponds [57,58]. Despite negative crowding effects, it is therefore expected that *Daphnia* will have a strong impact on *E*. *coli* in water. Recent work has shown that *Daphnia* effectively removed fine particulate matter during tertiary sewage treatment, holding promise for being a sustainable and efficient tool in faecal pollution treatment [59]. Although further studies are needed to confirm *Daphnia* contribution to faecal pollution treatment, our results illustrate the potential of the filter-feeder to clear *E*. *coli* from the water column.

### *Daphnia* impact on the fate of *E*. *coli* in freshwater

Since the early study of McMahon and Rigler that reported *E*. *coli* ingestion kinetics by *Daphnia* using radioisotopes [30], the impact of *Daphnia* on the fate of *E*. *coli* in water has not been addressed despite their co-occurrence in many freshwater resources (ex. lakes, reservoirs) affected by faecal pollution. Considering the role of *E*. *coli* as indicator of faecal pollution, it is essential to better understand its fate in presence of *Daphnia*. In this study, we showed that *Daphnia* was able to remove significant amounts of *E*. *coli* from the water and that *E*. *coli* initial concentration (Fig 2) and the presence of local biota (Fig 3) strongly influenced the overall impact of *Daphnia* on the fate of the faecal indicator. The contribution of *Daphnia* to *E*. *coli* loss rates decreased more than twice when using 1,000 fold lower *E*. *coli* concentrations. When assessed in a freshwater matrix, it decreased even more and required a *Daphnia* population of >32 ind.L^-1^ to overcome natural *E*. *coli* losses caused by matrix-related factors. It is therefore expected that *Daphnia* will essentially impact *E*. *coli* during population blooms, which can seasonally peak above 100 ind.L^-1^ in many freshwater habitats [42]. Under certain conditions (ex. high food amounts) *E*. *coli* could survive *Daphnia* gut passage and remain culturable (Figs 1b and 2) as has been shown for lake bacteria [48]. In nature though, the faecal indicator undergoes additional environmental stresses [3,4], which may reduce *E*. *coli* resistance to gut passage, but it remains to be tested using stressed *E*. *coli* cells. We hypothesize that, when occurring at sufficient densities, *Daphnia* could act as natural filter that removes *E*. *coli* from the water and seasonally improve microbial water quality in freshwater resources used for drinking water production and/or bathing.

Additional improvements can be done to the present experimental setup. Since we used a population of homogenously sized *Daphnia* individuals, it would be interesting to assess the impact of a heterogeneous population on *E*. *coli* loss rates. For instance, a mixed *D*. *pulex* population of varying body sizes could change the observed *E*. *coli* loss rates since filtration rates are related to body size [42,60]. Also, we used the cosmopolitan *Daphnia pulex* as model organism, but other *Daphnia* species such *D*. *magna*, which displays higher filtrations rates, should be assessed. Finally, simultaneous exposure of *E*. *coli* and faecal pathogens to *Daphnia* would enable to determine how the freshwater grazer comparatively affects their respective fate in water.

In conclusion, *Daphnia* significantly impacted the culturability and viability of *E*. *coli* in water. In lake water, *Daphnia* effect on *E*. *coli* loss rates increased with population densities and overcame natural *E*. *coli* losses at densities between 32 and 65 ind.L^-1^. During summer months in presence of sufficiently high population densities, *Daphnia* is thus likely to be a significant driver of *E*. *coli* fate in drinking water supplies and/or recreational water bodies.

## Supporting information

S1 Table Characterisation of zooplankton biota in the lake water matrix sampled at Missisquoi Bay (QC). (DOCX)
