# Supplementary material for: How does the cladoceran Daphnia pulex affect the fate of Escherichia coli in water?
Source: PLoS One. 2017 Feb 8;12(2):e0171705. doi: 10.1371/journal.pone.0171705 (PMC5298254; doi:10.1371/journal.pone.0171705)
Supplement: S1 Table — (DOCX) [file pone.0171705.s001.docx]

**Supporting information**

**S1 Table. Characterisation of zooplankton biota in the lake water matrix sampled at Missisquoi Bay (QC).**

| Zooplankton groups | | Population density^*^ | Dominant taxa (≥10 ind.L^-1^) |
| --- | --- | --- | --- |
| Metazooplankton | Cladocerans | 67 | *Chydoridea spp, Bosmina longirostris, Ceriodaphnia sp* |
|  | Rotifers | 202 | *Polyarthra sp, Keratella cochlearis* |
|  | Copepods | 55 | Nauplii |
| Protozooplankton | HNF | 868 | Not determined |

HNF, heterotrophic nanoflagellates

^*^ expressed in ind.L^-1^ and ind.mL^-1^ for metazooplankton and protozooplankton, respectively.
